# The Placebo-Controlled Effect of Percutaneous Coronary Intervention on Exercise Induced Changes in Anti-Malondialdehyde-LDL Antibody Levels in Stable Coronary Artery Disease: A Substudy of the ORBITA Trial

**DOI:** 10.3389/fcvm.2021.757030

**Published:** 2021-10-11

**Authors:** Adam Hartley, Matthew Shun-Shin, Mikhail Caga-Anan, Christopher Rajkumar, Alexandra N. Nowbar, Michael Foley, Darrel P. Francis, Dorian O. Haskard, Ramzi Y. Khamis, Rasha K. Al-Lamee

**Affiliations:** ^1^Department of Vascular Sciences, National Heart and Lung Institute, Imperial College London, London, United Kingdom; ^2^Department of Cardiovascular Trials and Epidemiology, National Heart and Lung Institute, Imperial College London, London, United Kingdom

**Keywords:** atherosclerosis, percutaneous coronary intervention (PCI), exercise, LDL-cholesterol, oxidised LDL (oxLDL), malondialdehyde-modified LDL (MDA-LDL)

## Abstract

**Aim:** Malondialdehyde-modified low-density lipoprotein (MDA-LDL) forms a significant component of oxidised LDL. The effects of exercise on levels of MDA-LDL and anti-MDA-LDL antibodies are not well-understood. Furthermore, it is not known whether these can be modified in patients with coronary artery disease by percutaneous coronary intervention (PCI).

**Methods:** The Objective Randomised Blinded Investigation with optimal medical Therapy of Angioplasty in stable angina (ORBITA) trial was the first blinded, multi-centre randomised trial of PCI vs. placebo procedure for angina relief. Serum samples were available at four time-points: pre-randomisation pre- (P1) and post- (P2) exercise and post-randomisation (6-weeks following the PCI or placebo procedure), pre- (P3) and post- (P4) exercise. ELISAs were performed using laboratory-developed assays for MDA-LDL (adjusted for Apolipoprotein B) and anti-MDA-LDL antibodies.

**Results:** One hundred ninety-six of the 200 patients (age 66.1 [SD 8.99] years, 28% female) with severe single vessel coronary artery disease suitable for PCI enrolled in the ORBITA trial had blood available for analysis. With exercise at pre-randomisation (P2–P1) there was no significant change in adjusted MDA-LDL (−0.001, 95% CI −0.004 to 0.001; *p* = 0.287); however, IgG and IgM anti-MDA-LDL significantly declined (−0.022, 95% CI −0.029 to −0.014, *p* < 0.0001; −0.016, 95% CI −0.024 to −0.008, *p* = 0.0002, respectively). PCI did not have a significant impact on either the pre-exercise values (P3 controlling for P1) of MDA-LDL (*p* = 0.102), IgG (*p* = 0.444) or IgM anti-MDA-LDL (*p* = 0.909). Nor did PCI impact the exercise induced changes in these markers (P4 controlling for P1, P2, and P3) for MDA-LDL (*p* = 0.605), IgG (*p* = 0.725) or IgM anti-MDA-LDL (*p* = 0.171). Pre-randomisation ischaemia on stress echo did not impact these interactions.

**Conclusions:** Exercise results in an acute reduction in anti-oxLDL antibodies in patients with severe single vessel coronary disease, possibly indicating an induction in homoeostatic clearance via the innate immune system. However, PCI did not ameliorate this effect.

## Introduction

Oxidised low-density lipoprotein (oxLDL) is found both within atherosclerotic plaques and the plasma of patients with cardiovascular disease (CVD). It has been shown, in observational studies, to be a significant predictor of cardiovascular outcome, reflecting a central role in atherogenesis (1–3).

Oxidation specific epitopes (OSEs) on oxLDL can act as “danger associated molecular patterns,” whereby they undergo recognition by members of the innate immune system such as C-reactive protein, complement proteins and both “natural” and “adaptive” antibodies (3). This provides an important homeostatic mechanism for oxLDL clearance (4). Anti-oxLDL antibodies of the IgM subclass have been generally found to confer protective benefit from coronary artery disease (CAD) and CVD, and indeed lower levels of IgM to one well-characterised epitope [malondialdehyde-modification of LDL (MDA-LDL)] have been linked with features of atherosclerotic plaques vulnerable to rupture (5). Conversely, the association of IgG anti-oxLDL antibodies with CVD is less clear (6).

Exercise, although of course widely accepted to carry significant health advantages, can transiently increase circulating oxLDL (7), and high levels of physical activity may counterintuitively contribute to atherogenesis in the presence of adequate substrate (8).

It has been demonstrated previously that percutaneous coronary intervention (PCI) can lead acutely to transiently higher levels of oxLDL in the plasma, attributed to either direct mechanical release/disruption from stented atherosclerotic plaque or increased generation (9). Whether or not this is clinically significant is unknown.

In this study we sought to assess the effects of exercise on plasma MDA-LDL in relation to anti-MDA-LDL antibodies. The availability of blood samples collected from CAD patients enrolled in the Objective Randomised Blinded Investigation with optimal medical Therapy of Angioplasty in stable angina (ORBITA) trial provided the unique opportunity of determining whether any changes with exercise we observed are influenced by coronary artery intervention (10).

## Methods

### Study Protocol

The ORBITA trial was a blinded, multi-centre randomised trial of PCI (105 patients) vs. a placebo procedure (95 patients) for stable CAD. The full ORBITA study protocol has been described previously (10). In brief, patients were enrolled if they had severe (≥70%) single vessel stenoses suitable for PCI. Key exclusion criteria were angiographic stenosis ≥50% in a non-target vessel, acute coronary syndrome, previous coronary artery bypass graft surgery, left main stem coronary disease, severe valvular disease or left ventricular systolic impairment. After enrolment, patients received 6 weeks of intense anti-anginal medication optimisation and were then randomised 1:1 to undergo PCI or a placebo procedure. The primary endpoint was difference in exercise time increment between baseline and 6-week follow-up between the two groups. Dobutamine stress echocardiography and cardiopulmonary exercise testing were performed at pre-randomisation after medication optimisation and 6-weeks post-randomisation, as previously described (11). From this a stress echo score was calculated (11); in brief, each abnormal segment was assigned a single point and segments totalled.

### Ethical Approval

The London Central Research Ethics Committee (13/LO/1340) approved the study and the investigation conformed to the principles outlined in the Declaration of Helsinki. Written consent was obtained from all patients prior to enrolment. The study is registered with ClinicalTrials.gov, number NCT02062593.

### Blood Samples and Exercise Protocol

**Figure 1:**
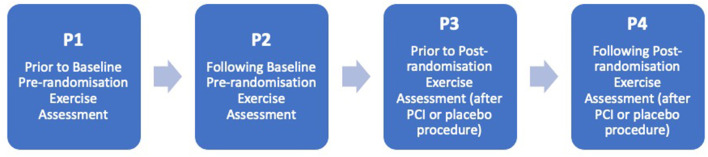
Timepoints of blood sampling throughout the study.

Blood samples were obtained on the day of pre-randomisation assessment (after the six-week medical optimisation run-in), prior to exercise (timepoint P1) and 3 h after exercise (P2). At post-randomisation assessment (6 weeks following the PCI or placebo procedure) further blood tests were obtained: before (P3) and 3 h after (P4) exercise (Figure 1). On both occasions exercise consisted of a cardiopulmonary exercise test, performed with the QUARK CPET breath-by-breath metabolic measurement system (COSMED, Rome, Italy). A physician and a physiologist, both blinded to treatment assignment, performed all tests. The test was continued until the development of limiting symptoms (angina, dyspnoea, or fatigue), heart rhythm or blood pressure abnormalities, or marked ST-segment deviation (≥0.20 mV associated with typical angina or in the first stage of exercise).

### Biomarker Assessment

Blood plasma specimens were stored at −80°C and thawed to room temperature prior to use. Enzyme-linked immunosorbent assays (ELISA) were performed to assess for MDA-LDL, IgG anti-MDA-LDL, IgM anti-MDA-LDL, and Apolipoprotein B (ApoB), as have been conducted previously (5, 12). ApoB levels were assessed as a marker for dilutional change. All researchers were blinded to treatment allocation. Intra-plate and inter-plate coefficients of variance were <5 and <15%, respectively.

#### MDA-LDL

A sandwich ELISA protocol was adopted using LO1, an in-house generated IgG monoclonal mouse autoantibody, to detect MDA-LDL (13). ELISA plates were coated with 10 μg/mL of LO1 as the capture antibody prior to washing and blocking with 3% bovine serum albumin (BSA) in phosphate-buffered saline (PBS). Goat biotinylated polyclonal anti-ApoB antibody (1:2000) (Abcam, Cambridge, MA, USA) and horseradish peroxidase (HRP)-conjugated streptavidin (R&D Systems, Minneapolis, MN, USA) at 1:200 dilution were used for detection. Subsequently, for this assay and all other assays, 3,3′,5,5′-tetramethylbenzidine (TMB) (Sigma Aldrich, Poole, UK) was added and the reaction stopped with 0.5M H_2_SO_4_. Plates were read at 450 nm optical density using a Synergy HT microplate reader (BioTek, VT, USA).

#### ApoB

ApoB was measured by ELISA in sandwich format. Plates were coated with polyclonal goat anti-human anti-ApoB (Abcam, Cambridge, MA, 1:2000). Detection was performed with goat biotinylated anti-ApoB antibody, prior to HRP-conjugated streptavidin and TMB as above.

#### IgG and IgM Anti-MDA-LDL

Acid hydrolysis of malondialdehyde bis (dimethyl acetal) (Sigma-Aldrich, Poole, UK) was performed to produce 0.5 M MDA solution, which was subsequently incubated with native LDL at 37°C for 3 h, as described previously (14). This generated MDA-LDL, which was then eluted through a Zeba Spin desalting column (ThermoFisher Scientific, Waltham, MA, USA), and PBS/0.01% EDTA added to prevent additional oxidation.

ELISA plates were coated with 10 μg/mL MDA-LDL, prior to washing and blocking as above. The primary detection antibodies were either unlabelled mouse anti-human IgG (Southern Biotech, Birmingham, AL, USA, 1:2000), or biotinylated mouse anti-human IgM (Southern Biotech, Birmingham, AL, USA, 1:2000). The secondary detection antibody was HRP-conjugated polyclonal rabbit anti-mouse antibody (Dako, Cambridgeshire, UK, 1:2000) for IgG anti-MDA-LDL and HRP-conjugated streptavidin for IgM anti-MDA-LDL.

#### Interpretation of ELISA Readouts

Concentrations of MDA-LDL and ApoB were derived by interpolation from log transformation of OD values onto a sigmoidal, four-parametric logistic curve using GraphPad Prism 8 (La Jolla, CA, USA). To correct for possible changes in plasma protein concentration due to exercise, each MDA-LDL concentration was adjusted by expressing the values as a ratio to ApoB concentration. MDA-LDL adjusted for ApoB is used throughout this study. Raw OD values were utilised for anti-MDA-LDL antibodies.

### Statistical Analysis

Continuous variables are presented as mean ± standard deviation (SD) or median with interquartile range (IQR), depending on the normality of the distribution. Categorical variables are presented as numbers with percentages.

Statistical analysis was performed using R 4.0.3 (R Foundation for Statistical Computing, Vienna, Austria) using the modelling package “rms” (15, 16). The change in biomarkers with exercise from timepoint P1 to P2 and P3 to P4 were assessed using paired Student's *t*-test. The remainder of the analyses were performed using ordinary least squares regression modelling. The effect of PCI vs. placebo on the pre-exercise (P3) biomarkers was assessed by including the treatment arm, and the P1 time point in the model. The effect of PCI on the post-exercise biomarker was assessed by including treatment arm and the P1, P2, and P3 timepoint in the model. The effect of stress echo score on the pre-randomisation post-exercise (P2) biomarker was assessed by including the P1 timepoint, and the stress echo score with a restricted cubic spline with 3 knots. The effect of stress echo score on the placebo-controlled impact of PCI on the post-exercise (P4) biomarker levels was again assessed using modelling, including the treatment arm and pre-randomisation stress echo score (with a restricted cubic spline) and their interaction, and timepoints P1, P2, and P3.

## Results

### Baseline Characteristics

The ORBITA trial randomised 200 patients to either PCI or placebo procedure between January 6, 2014, and August 11, 2017. From these patients, 196 had blood samples available for analysis. Table 1 displays baseline patient characteristics. The mean age in the PCI group was 66.0 (9.50) (mean [standard deviation, SD]) years and 66.2 (8.45) years in the placebo group. There were no substantial differences in baseline demographics between the PCI and placebo groups. At pre-randomisation assessment, 98% (192/196) of patients were taking aspirin and 95% (186/196) were taking a statin. 78% (153/196) of the study population were taking β-blockers whilst 91% (178/196) were taking calcium channel antagonists. The median number of anti-anginal medications was three prior to randomisation.

**Table 1:** Baseline patient characteristics for the study population.

	**PCI (*n* = 102)**	**Placebo (*n* = 94)**	**All patients (*n* = 196)**
Age (years, SD)	66.0 (9.50)	66.2 (8.45)	66.1 (8.99)
Male (%)	71 (70%)	71 (76%)	142 (72%)
Hypertension (%)	70 (69%)	65 (69%)	135 (69%)
Hypercholesterolaemia (%)	79 (77%)	62 (66%)	141 (72%)
Previous myocardial infarction (%)	5 (5%)	7 (7%)	12 (6%)
Previous PCI (%)	9 (9%)	15 (16%)	24 (12%)
Diabetes Mellitus (%)	15 (15%)	21 (22%)	36 (18%)
Current smoker (%)	10 (10%)	15 (16%)	25 (13%)
Angina duration (months, SD)	9.7 (15.9)	8.4 (7.6)	9.1 (12.6)
**Left ventricular systolic function**			
- Normal (%)	96 (94%)	84 (89%)	180 (92%)
- Mildly impaired (%)	3 (3%)	7 (7%)	10 (5%)
- Moderately impaired (%)	3 (3%)	3 (3%)	6 (3%)
**Significant coronary stenosis**			
- LAD (%)	69 (68%)	65 (69%)	134 (68%)
- D1 (%)	2 (2%)	2 (2%)	4 (2%)
- Intermediate (%)	1 (1%)	2 (2%)	3 (2%)
- LCx (%)	9 (9%)	10 (11%)	19 (10%)
- OM1 (%)	4 (4%)	0 (0%)	4 (2%)
- RCA (%)	17 (17%)	15 (16%)	32 (16%)

*PCI, percutaneous coronary intervention; LAD, left anterior descending; D1, 1st diagonal; LCx, circumflex; OM1, 1st obtuse marginal; RCA, right coronary artery*.

### Effect of Exercise on Baseline MDA-LDL and Anti-MDA-LDL Antibodies

**Figure 2:**
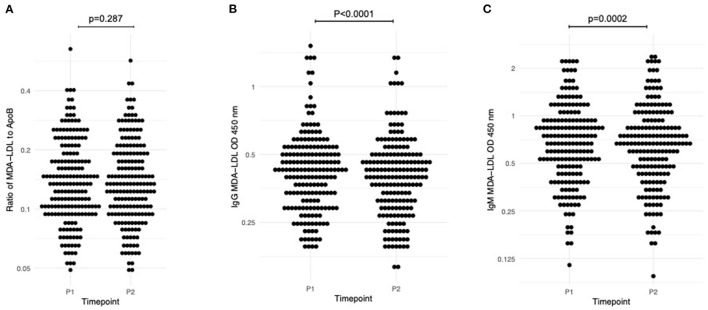
Dynamic change in measured biomarkers between P1 (baseline pre-randomisation assessment, pre-exercise) and P2 (baseline pre-randomisation assessment, post-exercise). **(A)** Adjusted MDA-LDL; **(B)** IgG anti-MDA-LDL; **(C)** IgM anti-MDA-LDL. One sample *t*-test used to assess significance.

Table 2 displays the average values for adjusted MDA-LDL and anti-MDA-LDL antibodies at each study timepoint. There was no significant change with exercise (P1–P2) in ApoB (−9.189 OD, 95% CI −19.913 to 1.542; *p* = 0.09) or in MDA-LDL adjusted for ApoB levels (−0.001, 95% CI −0.004 to 0.001; *p* = 0.287; Figure 2A). On the other hand, there was a significant reduction in anti-MDA-LDL antibodies with exercise: IgG anti-MDA-LDL declined by −0.022 OD (95% CI −0.029 to −0.014; *p* < 0.0001; Figure 2B; whilst IgM anti-MDA-LDL reduced by −0.016 OD (95% CI −0.024 to −0.008; *p* = 0.0002; Figure 2C).

**Table 2:** Average biomarker values at each study timepoint (P1–P4).

	**P1 (*n* = 188)**	**P2 (*n* = 186)**	**P3 (*n* = 186)**	**P4 (*n* = 183)**
MDA-LDL	0.155 (0.085)	0.154 (0.083)	0.158 (0.116)	0.160 (0.123)
IgG anti-MDA-LDL	0.454 (0.214)	0.432 (0.198)	0.473 (0.223)	0.467 (0.216)
IgM anti-MDA-LDL	0.773 (0.457)	0.759 (0.460)	0.832 (0.517)	0.808 (0.492)

*MDA-LDL is expressed as a ratio of MDA-LDL to Apolipoprotein B; anti-MDA-LDL antibodies are expressed as optical density values at 450 nm. Mean (standard deviation)*.

### Effect of PCI vs. Placebo on Pre-exercise and Post-exercise MDA-LDL and Anti-MDA-LDL Antibodies

PCI, when compared to placebo, did not have a significant impact on pre-exercise (P3 controlling for P1) adjusted MDA-LDL, IgG or IgM anti-MDA-LDL (*p* = 0.102, *p* = 0.444, *p* = 0.909 respectively). Similarly, PCI did not have a significant impact on post-exercise (P4 controlling for P3, P2, and P1) biomarker measurements (adjusted MDA-LDL *p* = 0.605, IgG anti-MDA-LDL *p* = 0.725, IgM anti-MDA-LDL *p* = 0.171). This is despite the exercise induced changes in the measured antibodies remaining in the whole study population when retested, with significant reductions in both IgG and IgM anti-MDA-LDL (−0.006, 95% CI −0.013 to −0.0004, *p* = 0.036; −0.015, 95% CI −0.024 to −0.006, *p* = 0.002, respectively. Again, there was no significant change in adjusted MDA-LDL (0.0007, 95% CI −0.003 to 0.004; *p* = 0.707.

We also examined for a relationship between oxidative biomarker change with exercise at baseline (P2 controlling for P1) and exercise intensity. There were no significant associations between MDA-LDL (*p* = 0.355), IgG (*p* = 0.386) or IgM (*p* = 0.657) anti-MDA-LDL antibodies with exercise duration. Similarly, there were no relationships between biomarker changes and maximum oxygen consumption (VO_2_ max) (MDA-LDL [*p* = 0.121], IgG anti-MDA-LDL [*p* = 0.701], IgM anti-MDA-LDL [*p* = 0.07]).

Of the patients included in the study, 180 had dobutamine stress echocardiography performed at baseline. The pre-randomisation stress echo score (with higher scores reflecting more abnormal myocardial segments) did not significantly affect the post-exercise (P2 controlling for P1) MDA-LDL (*p* = 0.349), IgG anti-MDA-LDL (*p* = 0.852) or IgM anti-MDA-LDL (*p* = 0.255). Furthermore, the pre-randomisation stress echo score did not affect the placebo-controlled impact of PCI on the post-exercise (P4) levels of adjusted MDA-LDL (*p* = 0.484), IgG anti-MDA-LDL (*p* = 0.750) or IgM anti-MDA-LDL (*p* = 0.759). Therefore, even patients with high levels of baseline ischaemia saw no significant effect of PCI on exercise-induced biomarker change.

## Discussion

In this study we find that high intensity exercise resulted in significant plasma oxidative biomarker changes in patients with severe CAD on intensively up-titrated anti-anginal medical therapy, with IgG and IgM anti-MDA-LDL antibodies significantly decreasing following exercise. PCI neither significantly influenced the biomarkers at baseline prior to exercise, nor their changes after exercise.

One explanation for the immune system modulation in this population is that the vigorous exercise acts as a severe cellular stressor, resulting in oxidative stress with increased free radical generation and MDA-LDL formation. Consequently, there is consumption of the circulating anti-oxLDL antibodies as they beneficially clear the increased antigenic load, forming complexes and trafficking them to the reticuloendothelial system for removal in a homeostatic clearance mechanism. These observations are consistent with other studies from our laboratory, which demonstrate acute reduction in anti-MDA-LDL antibodies following major vascular surgery and coronary artery bypass grafting (17).

The degree of myocardial ischaemia as assessed by stress echocardiography did not influence biomarker changes in this study. We also report no significant change in MDA-LDL, which possibly may be due to rapid immune clearance. Nonetheless, an acute increase in circulating oxLDL with exercise has been demonstrated previously in diseased populations, such as hypertensive hypercholesterolaemic patients (18), chronic heart failure (19), diabetes mellitus (20) and in aged populations (7, 21). A possible explanation for this discrepancy is the timing when samples are obtained; blood samples were taken 3 h following exercise in this study, rather than immediately post exercise as in many of these studies. As such, the acute rise prior to homeostatic clearance may be missed at the time of blood sampling. Another possible explanation is that the highly optimised medical therapy renders the patients relatively non-ischaemic, and as such the biomarkers behave as though they were not sampled from patients with severe CAD. Indeed, there is no consensus in the literature on exercise-induced oxLDL changes in healthy participants (22–27), and in some circumstances no changes in oxLDL levels, even following very high intensity exercise, have been found (28).

The chronic effect of regular exercise on baseline oxLDL levels has been examined, with studies reporting improved oxidised lipid profiles with sufficiently robust exercise (29–33). Moreover, exercise training has been demonstrated to counteract raised baseline oxLDL levels that are found in overweight or obese individuals (34), to combat the acute exercise-induced increased LDL oxidation that occurs with advancing age (21), and in patients with CVD undergoing cardiac rehabilitation, those who completed the 6-month exercise program had significantly lower baseline MDA-LDL levels than their counterparts (35). Additionally, MDA-LDL levels have been shown to vary inversely proportionally to daily pedometer step counts (36). Moreover, in animal models of exercise, it has been demonstrated that exercise can reduce oxidative stress (37) and even selectively increase the B-1 cell population and natural IgM levels (38).

One hypothesis to explain the apparent paradox between acute and chronic exercise and circulating oxLDL level is that recurrent plasma oxLDL exposure leads to greater homeostatic immune system induction. With a greater circulating anti-oxLDL antibody reservoir, there may have a better adapted clearance mechanism to cope with the oxidative stress of exercise once the antioxidant capacity is exceeded and go some way to explain why baseline oxLDL levels decline with greater fitness. Perhaps greater immune system induction could also underpin some of the cardiovascular benefit associated with exercise. Indeed, exercise-related cardiovascular events occur more commonly in inactive people with multiple cardiovascular risk factors than in well-trained athletes, who may have more honed innate immunity (39). Immunosenescence, ageing of the adaptive and innate immune systems, may also explain the greater LDL oxidation that occurs with exercise in advancing age (7, 40).

In our study we only measured samples at 3 h following single episodes of exercise and identified immune consumption. If samples were tested at multiple earlier timepoints after frequent exercise we may expect to see a rebound rise in anti-oxLDL antibodies, as has been reported in the literature (41, 42). However, in a recent study by Bachi and colleagues, trained athletes after running a marathon exhibited no change in IgM or IgG anti-MDA-LDL antibodies immediately or 72-h after the race, despite increases in plasma oxLDL (27). Again, perhaps the timepoints tested missed any small dynamic changes with the utilised assay, or the elite fitness of the participants means that their baseline anti-oxLDL antibody levels are already fully induced.

There is some plausibility to the theory of immune induction following an oxLDL stimulus: using PCI as an example (43), studies have shown long-lasting immune system induction to rapid oxLDL increases, with raised IgG and IgM anti-oxLDL antibodies present out to 6 months following the index procedure (9, 44). Whilst these studies demonstrated immunomodulation following PCI, we did not find such a trend in this study, with unchanged baseline or post-exercise oxidative biomarkers. It may be expected that PCI, through restoring unobstructed flow and therefore coronary perfusion, would attenuate the immune system induction seen with exercise. As above, this unexpected result may be due to sample timing (taken at 6-weeks following the procedure, where the peak level may have been missed), or an interaction with medical therapy, with a very high proportion of patients on a statin and well-titrated anti-anginal therapy. What is demonstrated however, given the lack of association between severity of baseline ischaemia and dynamic biomarker change with exercise, is that anti-MDA-LDL antibodies in themselves were not biomarkers of ischaemia *per se*, in this study.

### Limitations

The main limitations of this study lie in the fixed timing of blood samples for biomarker analysis. The availability of plasma samples immediately following exercise and the PCI/placebo procedure would shed further light onto the interaction of the stimuli and the immune system. Furthermore, the limited number of blood sampling timepoints makes it difficult to exactly elucidate when plasma levels peak and when they are cleared. Another caveat of this study is that MDA adduction of LDL is but one of a range of possible post-translational oxidative modifications on LDL; however, MDA-LDL does comprise a significant component of the oxLDL population (45).

## Conclusion

Using blood samples obtained from the ORBITA trial, this study demonstrates that exercise results in an acute reduction in anti-oxLDL antibodies, possibly indicating an induction in homoeostatic clearance of oxLDL via the innate immune system. However, PCI did not ameliorate this effect.

## Data Availability Statement

The raw data supporting the conclusions of this article will be made available by the authors upon appropriate request following review by the corresponding author.

## Ethics Statement

The studies involving human participants were reviewed and approved by London Central Research Ethics Committee (13/LO/1340). The patients/participants provided their written informed consent to participate in this study.

## Author Contributions

AH, MC-A, and RA-L: investigation. DH, DF, RA-L, and RK: supervision. AH, MC-A, and MS-S: data analysis. AH: writing—original draft. AH, MS-S, CR, AN, MF, DH, RK, and RA-L: writing—review and editing. All authors contributed to the article and approved the submitted version.

## Funding

AH was funded by a Wellcome Trust Clinical Research Fellowship (220572/Z/20/Z). RK was funded by a BHF Clinical Research Fellowship (FS/17/16/32560) and had received a Wellcome Trust Clinical Research Fellowship (095034/Z/10/Z) (as part of the Wellcome Trust/GSK Fellowship programme). DH received professorial chair funding from the British Heart Foundation (BHF). AN was supported by the NIHR Academy.

## Conflict of Interest

The authors declare that the research was conducted in the absence of any commercial or financial relationships that could be construed as a potential conflict of interest.

## Publisher's Note

All claims expressed in this article are solely those of the authors and do not necessarily represent those of their affiliated organizations, or those of the publisher, the editors and the reviewers. Any product that may be evaluated in this article, or claim that may be made by its manufacturer, is not guaranteed or endorsed by the publisher.

## Abbreviations

ApoB: apolipoprotein B-100
BSA: bovine serum albumin
ELISA: enzyme-linked immunosorbent assay
LDL: low-density lipoprotein
LDS: lithium dodecyl sulphate
MDA: malondialdehyde
MDA-LDL: malondialdehyde-conjugated LDL
OD: optical density
OxLDL: oxidised low-density lipoprotein
RT: room temperature.
